# Effect of Peroxyl Radical-Induced Oxidation on Functional and Structural Characteristics of Walnut Protein Isolates Revealed by High-Resolution Mass Spectrometry

**DOI:** 10.3390/foods11030385

**Published:** 2022-01-28

**Authors:** Xuechun Zhang, Xi Yang, Yunqian Li, Zhenxing Wang, Xuemei He, Jian Sun

**Affiliations:** 1 Guangxi Key Laboratory of Fruits and Vegetables Storage-Processing Technology, Nanning 530007, China; xuechun_zhang@163.com; 2 Institute of Environmental Remediation and Human Health, Southwest Forestry University, Kunming 650224, China; wangzhenxingfood@163.com; 3 Guangxi Academy of Agricultural Sciences, Nanning 530007, China; 4 College of Life Sciences, Southwest Forestry University, Kunming 650224, China; xi_yang2076@163.com (X.Y.); 18388173746@163.com (Y.L.)

**Keywords:** walnut protein isolates, oxidation, structural characteristics, functional properties, proteomics

## Abstract

The present study aims to investigate the structural and functional properties of oxidated walnut protein isolates (WPI) by 2,2′-azobis (2-amidinopropane) dihydrochloride (AAPH). The oxidation degree, changes in structural characteristics, processing properties, and protein modifications of WPI were measured. The results showed that oxidation significantly induced structural changes, mainly reflected by the increasing carbonyl content, and decreasing sulfhydryl and free amino groups. Moreover, the secondary structure of WPI was altered in response to oxidation, and large aggregates formed through disulfide cross-linking and hydrophobic interactions. Almost all the property indicators were significantly decreased by oxidation except the foaming property and water/oil holding capacity. Mass spectrometry analysis showed that 16 different modifications occurred in amino acid side chains, and most of the protein groups with higher numbers of modifications were found to be associated with allergies, which was further confirmed by the reduction in antigenicity of the major allergen (Jug r 1) in WPI. Meanwhile, we used oxidation-related proteins for gene ontology (GO) enrichment analyses, and the results indicated that 115, 204 and 59 GO terms were enriched in terms of biological process, molecular function, and cellular component, respectively. In conclusion, oxidation altered the groups and conformation of WPI, which in turn caused modification in the functional properties correspondingly. These findings might provide a reference for processing and storage of walnut protein foods.

## 1. Introduction

Walnut (*Juglans regia* L.) is one of the common tree nuts widely grown worldwide. The planted area and production of walnuts in China ranks first in the world [1]. It has been reported that walnut kernels are rich in unsaturated oils, protein, vitamins, minerals and some bioactive compounds which confer antioxidant, cholesterol reduction, arterial improving, immunomodulatory and other biological activities [2,3]. Therefore, walnut kernels are widely consumed in China (409,608 metric tons in 2019) and even worldwide (914,383 metric tons in 2019) for their health promotion and delicious taste [4].

In view of the high-cholesterol and saturated fatty acids content in animal-derived foods, plant proteins are gaining increasing interest for their specific advantages. Soy protein, peanut protein and walnut protein, etc. have been studied for their nutritional, processing and physiological properties. Among them, as one of the main by-products of the oil industry, walnut protein has great potential of high value utilization. Currently, there have been gradually increasing studies on walnut protein due to its large amount of consumption, such as classification and characterization [5], characteristics analysis and functional properties modification [3,6,7,8,9].

Oxidation is one of the three major chemical reactions. In contrast to other forms of modification, oxidation can occur during the harvesting, storage and processing of foods [10]. It has been reported that radical-induced oxidation could change the properties, conformation, digestibility and allergenicity of food proteins [11,12,13,14]. As we know, walnut kernels contain high amounts of unsaturated fatty acids, which could generate radical intermediates and secondary products through oxidative stress, and then induce the oxidized walnut protein, in turn, to undergo corresponding changes.

Peroxy radical (ROO·) is an important radical during the lipid oxidation process. However, the effect of peroxy radical on the properties and structure of walnut protein remains unclear. Thus, in this study, walnut protein isolates (WPI) were prepared and oxidized by 2,2′-azobis (2-amidinopropane) dihydrochloride (AAPH), a water-soluble azo-free radical initiator, and then, the effect of peroxy radical on the main groups, structure, functional properties, and antigenicity of WPI were investigated. Meanwhile, the oxidation site and modifications of WPI were analyzed by proteomics technology. This study hopes to provide advice on quality control and utilization of walnut protein.

## 2. Materials and Methods

### 2.1. Materials and Reagents

Walnut kernels were purchased from the local market in Baoshan, China, 2,2′-azobis (2-amidinopropane) dihydrochloride (AAPH), 2,4-dinitrophenylhydrazine (DNPH), 5,5’-Dithiobis-(2-nitrobenzoic acid) (DTNB) and 8-Anilino-1-naphthalenesulfonic acid (ANS) were bought from Sigma-Aldrich Co. LLC. (Shanghai, China), and double antibody sandwich ELISA Kits was bought from Shanghai Meilian Biotechnology Co., Ltd. (Shanghai, China). All other reagents used were analytically pure, and water was distilled water in this study.

### 2.2. Walnut Protein Isolates (WPI) Preparation

WPI was extracted by the alkali dissolution-acid precipitation method according to Qin et al. [9] with slight modifications. Walnut kernels were pulverized and degreased with six-fold volumes of hexane three times. After being dried in a fume hood at room temperature, 25-fold volumes of sodium hydroxide solution (pH 8.7) were added to the defatted walnut powder, stirring the extract for 1 h at 48 °C, followed by centrifugation at 10,000× *g* for 20 min. The supernatant was adjusted to pH 5, and centrifuged using the same conditions as described above. WPI was obtained by washing the precipitate with H_2_O until neutral and freeze-drying it at −80 °C for 24 h. The WPI samples were stored frozen at −80 °C for spare use.

### 2.3. Oxidation of WPI

The oxidation of WPI was performed according to Duan et al. [15] with some modifications. Briefly, dried WPI was dispersed in phosphate buffer (PB, 0.01 mol/L, pH 7.4) to prepare 25 mg/mL protein suspension, then mixed with various concentrations of AAPH (0, 0.04, 0.20, 1.00, 3.00, 5.00 and 10.00 mmol/L), followed by incubation for 24 h at 37 °C under light protection. The reaction solutions were centrifuged for 30 min at 1000× *g* and 4 °C. The supernatants were dialyzed (molecular weight cut-off 7 kDa) against distilled water for 72 h at 4 °C. Next, the dialysates were freeze-dried at −80 °C for 24 h to obtain AAPH oxidized WPI (APPH-WPI) and were stored frozen at −80 °C for spare use.

### 2.4. Determination of Oxidation Degree of AAPH-Oxidized WPI (APPH-WPI)

#### 2.4.1. Determination of Carbonyl Content

The carbonyl contents of WPI and APPH-WPI were measured by 2,4-dinitrophenylhydrazine (DNPH) assay. Briefly, 1.5 mL protein dispersion (10 mg/mL) was mixed with 1 mL DNPH solution (10 mmol/L, containing 2 mol/L HCl), after incubation in the dark for 1 h at room temperature, then 1 mL of trichloroacetic acid (TCA, 20%) was added and it was mixed well, followed by centrifugation at 8000× *g* for 15 min. The precipitate was washed with 1 mL of ethyl acetate-ethanol (1:1, *v/v*) three times, and then dissolved in 4 mL urea (6 mol/L) for 20 min at 37 °C. After centrifugation at 8000× *g* for 15 min, absorbance of the supernate was read at 370 nm by a microplate reader (Biotek, Winooski, VT, USA), and the carbonyl content of protein was expressed as nmol/mg protein using the molar extinction coefficient of 22,000 L∙mol^−1^∙cm^−1^ [16]. Measurement was repeated at least three times to calculate the carbonyl content of the samples.

#### 2.4.2. Determination of Sulfhydryl Group Content

The sulfhydryl group content of WPI and APPH-WPI was measured in triplicate and repeated three times by using the DTNB assay. A 4 mg/mL DTNB solution was first prepared in 0.086 M Tris-Gly buffer (containing 5 mM EDTA, pH 8.0), followed by a suspension of protein samples in Tris-Gly buffer (containing 2% (*w/w*) sodium dodecyl sulfate (SDS)) to obtain a concentration of 10 mg/mL. Then, 1 mL of the sample solution was reacted with 10 μL of DTNB solution for 1 h at room temperature, followed by centrifugation at 10,000× *g* for 10 min, the absorbance of supernate was then measured at 412 nm against the blank (without DTNB and sample). The free sulfhydryl group contents of protein samples were calculated using the extinction coefficient of 13,600 L∙mol^−1^∙cm^−1^, while the total sulfhydryl content was measured as a similar procedure, except that 8 M urea was added to the sample buffer [17,18].

#### 2.4.3. Determination of Free Amino Groups Content

The o-phthaldialdehyde (OPA) method was used to measure the free amino groups content of proteins samples. OPA was first dissolved in methanol solution (0.4 g/mL), followed by addition of 2.5 mL of SDS solution (200 g/L), 25 mL of boric acid solution (0.1 mol/L), and 100 μL of β-mercaptoethanol. The mixture was diluted to a certain volume with distilled water to obtain the OPA working solution for the subsequent analysis. Next, an amount of 200 μL of protein solution was mixed with 4 mL of OPA working solution, and then water bathed at 35 °C for 2 min. The absorbance at 340 nm was measured against distilled water as control. This trial was repeated three times to calculate the free amino group content [19].

### 2.5. Determination of Structural Characteristics of APPH-WPI

#### 2.5.1. Particle Size Distributions

The particle size distribution of protein samples was determined by laser scattering particle size distribution analyzer (Partica, LA-960, Kyoto, Japan) with the relative refractive index and absorption set as 1.414 and 0.001, respectively. Briefly, the protein samples were suspended in distilled water to obtain a 5 mg/mL solution, and then the particle size distribution and the mean diameter of sample solutions were assessed and repeated at least three times, and then made into graphs [20].

#### 2.5.2. Sodium Dodecyl Sulphate–Polyacrylamide Gel Electrophoresis (SDS-PAGE) Analysis

SDS-PAGE was performed under non-reducing and reducing conditions according to the previous reports [21,22]. Briefly, an amount of 15 μL of sample solution (1 mg/mL) was mixed with 5 μL of 4 × protein loading buffer, followed by heating in a 100 °C water bath for 5 min, and then immediately placed in an ice bath. Next, the sample solutions were centrifuged (10,000× *g*, 5 min) and a 10 μL portion of supernatant was taken for electrophoresis (Bio-rad, Powerpac Basic, Hercules, CA, USA) using 12% separating gel and 5% stacking gel, with standards of known molecular weight ranging from 10 to 200 kDa. The gels were decolored and photographed for further analysis. This experiment was repeated with three different batches of samples.

#### 2.5.3. Intrinsic Fluorescence

The protein sample was dispersed in PB (0.01 mol/L, pH 7.9) to prepare a solution at 2.5 µg/mL, followed by centrifugation for 10 min at 10,000× *g*, 4 °C. The fluorescence intensity of supernate was scanned three times (Thermo Fisher Scientific, Lumina, Waltham, MA, USA) from 290 to 500 nm with excitation at 283 nm [23].

#### 2.5.4. Surface Hydrophobicity (H_0_)

The surface hydrophobicity (H_0_) of WPI was determined by the 1-anilino-8-naphthalenesulfonic acid (ANS) fluorescence probe method [24]. Each protein sample was diluted to 0.005, 0.01, 0.02, 0.05, 0.1, 0.2 and 0.5 mg/mL in PB (10 mM), respectively, then a 2 mL portion of the aforementioned solution was mixed well with 20 μL ANS solution (in 10 mM PB) and allowed to stand for 3 min at room temperature. The fluorescence intensity was measured at 390 nm excitation wavelength and 470 nm emission wavelength. Next, a linearity curve was drawn with the protein concentration as the abscissa and fluorescence intensity as the vertical coordinate. The slope of the curve was calculated as surface hydrophobicity of protein sample. This experiment was repeated with three different batches of samples.

#### 2.5.5. Fourier Transformed Infrared Spectroscopy (FT-IR)

Protein powder samples were mixed with potassium bromide and pressed into a thin tablet, followed by full-band scanning from 4000 to 400 cm^−1^ using a Fourier transform infrared spectrometer (Thermo Fisher Scientific, Nicolet IS50, Waltham, MA, USA), and scans were averaged three times for each spectrum. The data were analyzed and fitted with Peak fit 4.12 and OMNIC 8.2 software to obtain the proportions of the secondary structure (α-helix, β-sheet, β-turn, and random coil), respectively [15].

### 2.6. Determination of Physicochemical Properties of APPH-WPI

#### 2.6.1. Solubility

A protein sample was suspended in distilled water to obtain a final solution at 0.02 mg/mL and stirred for 1 h at room temperature, followed by centrifugation (6500× *g*, 4 °C) for 20 min. The protein content of supernate was measured by the micro-Kjeldahl method, each replicate was determined three times, and the solubility was calculated as the percentage of the soluble protein (g) compared to total protein sample (g) [5].

#### 2.6.2. Foaming ability (FA) and Foaming Stability (FS)

FA and FS of protein samples were measured according to Qian and Sun [8] with slight modification. A protein sample dispersion (1 mg/mL) was prepared by being suspended in PB (0.05 mol/L, pH 7.0), and after stirring for 1 h at room temperature, a 40 mL portion of the dispersion was homogenized at 10,000 rpm for 2 min. The volume of stirring solution at the first moment (0 min) and 60 min were recorded and calculated according to the follow equations to obtain FA and FS. This experiment was repeated with three different batches of samples.

FA(%) = (V_1_ − V)/V (1)

FS(%) = (V_2_ − V)/(V_1_ − V) (2)

where V_1_ is the volume of sample solution homogenized at the first moment, V is the initial volume of sample solution (40), and V_2_ is the volume of sample solution kept for 30 min after being homogenized.

#### 2.6.3. Water/Oil Holding Capacity (WHC/OHC)

An amount of 200 mg protein powder was mixed well with 10 mL water or soybean oil for 1 min in a weighted centrifuge tube using a vortex; afterward, the mixture was centrifuged at 3000× *g* for 20 min and the supernate was discarded, while the pellet was weighed. This trial was repeated three times and the WHC/OHC was expressed as the amount of absorbed water/oil (g) per gram protein sample [25].

### 2.7. Mass Spectrometry Analysis

Mass spectrometry analysis was carried out by nano liquid chromatography-Q Exactive (LC-QE) mass spectrum (Thermo Fisher Scientific, Q Exactive, Waltham, MA, USA). The control and oxidized WPI (10 mmol/L AAPH) samples were prepared by reduction and alkylation methods, followed by trypsin-hydrolyzed (1:50, *w/w*) at 37 °C for 20 h. The hydrolysates were lyophilized and re-dissolved in formic acid solution (FA, 0.1%, *w/w*) after desalting, and stored at −20 °C for further liquid chromatography (LC) separation. The LC conditions were as follows: Solution A, aqueous solution of 0.1% FA, solution B, 84% acetonitrile containing 0.1% FA. The hydrolysate was loaded into the trap column by automatic sampler after being balanced, then the full scan was performed and 20 fragment profiles were collected to obtain the mass charge ratios of peptides and peptide fragments. The mass spectrometry data were searched against the UniProtKB database by proteome discoverer software (Thermo Fisher Scientific, Version 1.4, Waltham, MA, USA).

### 2.8. Double Antibody Sandwich Enzyme-Linked Immunosorbent Assay (ELISA)

The allergenicity of WPI was evaluated by determining the content of Jug r 1, the major allergen of walnut, using double antibody sandwich ELISA Kits. WPI dispersions oxidized with 0, 5, 10, 15, and 30 mmol/L AAPH were prepared (3 mg/mL) and centrifuged at 5000× *g*, 4 °C for 30 min, and the supernatants were saved as sample solutions. A 50 μL portion of standard and mentioned sample solution were loaded onto the microplate wells, respectively, followed by being closed and incubated at 37 °C for 30 min, then the liquid was discarded and the microplates were washed 5 times by wash solution and patted dry. Next, enzyme-labeled reagent (50 μL) was added to each well except the blank well, after incubation and washing, and the chromogenic agents A (50 μL) and B (50 μL) were added to each well, mixed well and incubated in the dark at 37 °C for 10 min. Finally, the reaction was terminated by adding a stop solution (50 μL) to each well, and the absorbance at 450 nm was read (TECAN, F50, Männedorf, Switzerland). A standard curve was drawn with the standard concentration as abscissa and OD as the ordinate. This experiment was repeated with three different batches of samples, and the Jug r 1 content was calculated by the standard curve according to the corresponding OD.

### 2.9. Statistical Analysis

All experiments were repeated three times, and the results were expressed as average ± standard deviation (SD). The significance was determined by one-way ANOVA, and the graphs were drawn using origin 2021 software (Origin Lab, Northampton, MA, USA).

## 3. Results and Discussion

### 3.1. Oxidative Indicators Analysis

#### 3.1.1. Carbonyl Groups

The process of peroxy radical oxidation of protein is complex and accompanied by corresponding products generation, among which carbonyl group is the most widely used indicator for preliminary evaluation of oxidation degree [10]. The effect of peroxy radical on carbonyl content of WPI is shown in Table 1. Carbonyl content of oxidized WPI was significantly (*p* < 0.05) increased with the concentration of AAPH, and a maximum (1.48 nmol/mg) at 10 mmol/L AAPH was observed. As reported by previous studies [26], peroxy radicals were generated linearly with the decomposition of AAPH, in turn attacking the main peptide chain or side chain of WPI, and thus carbonyl derivatives such as aldehydes and ketones were formed accordingly. These results were similar to that of AAPH oxidized casein [27] and ovalbumin [17].

#### 3.1.2. Sulfhydryl Groups

Cysteine residues are sensitive to oxidation, and are susceptible to corresponding modification, therefore, the degree of protein oxidation can be reflected by the content of sulfhydryl groups. As shown in Table 1, with the increase of AAPH concentration, the total and free sulfhydryl groups of WPI decreased significantly (*p* < 0.05), and the minimums were achieved (1.25 and 114.26 nmol/mg) respectively when AAPH concentration was 10 mmol/L. This result is similar to that of peroxyl radical oxidized chickpea protein reported by Zhu et al. [23], the sulfhydryl groups were oxidized to both disulfide bonds and irreversible sulfonic and sulfonic acid groups by radicals [28]; moreover, other covalent cross-linking might have occurred during oxidation [18].

#### 3.1.3. Free Amino Groups

As depicted in Table 1, the free amino groups of oxidized WPI decreased with the AAPH concentration, and the minimum (0.31 nmol/mg) was discerned at a concentration of 10 mmol/L, which could be due to the oxidation of amino-containing side chain (threonine, proline, arginine and lysine residues) in WPI [29], and the formation of Schiff base with carbonyl groups by a covalent reaction [19]. This finding echoed that of the carbonyl result mentioned above and a similar result was obtained with oxidized *Coregonus peled* myofibrillar proteins [29].

### 3.2. Structural Characteristics Analysis

#### 3.2.1. Endogenous Fluorescence

**Figure 1:**
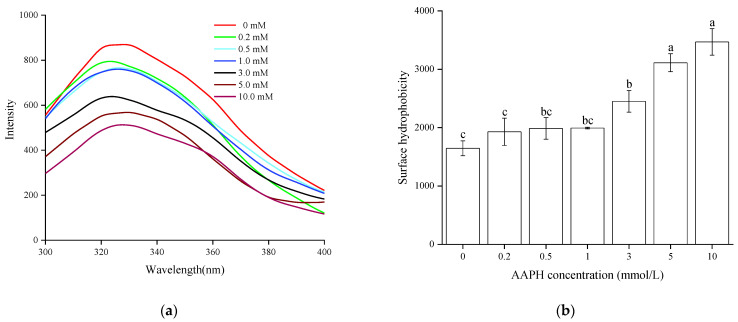
Endogenous fluorescence spectra (**a**) and surface hydrophobicity (**b**) of WPI oxidized with different concentration of AAPH. WPI = walnut protein isolates, AAPH = 2, 2′-azobis (2-amidinopropane) dihydrochloride. Different letters indicate significant differences between columns (*p* < 0.05).

The endogenous fluorescence of proteins originates from the emission of tryptophan residues, and the polarity of the micro-environment in which can be reflected by the changes of fluorescence peak. Therefore, endogenous fluorescence was often used to indicate the transformation of protein conformation. The effects of peroxy radical on the endogenous fluorescence intensity and maximum fluorescence emission wavelength of WPI are shown in Figure 1a. With increasing AAPH concentration, the fluorescence intensity of WPI showed a downward trend. This might be attributed to the unfolding of the WPI tertiary structure, and the tryptophan residue is oxidized to kynurenine, thus inducing a reduction in the endogenous fluorescence intensity. In addition, a blue shift of maximum fluorescence peak due to oxidation was also observed in Figure 1a, which is similar to the report by Zhang et al. [18]; hence, it was concluded that the tryptophan residue of WPI was exposed to hydrophobic environment which in turn made hydrophobic interactions by oxidation.

#### 3.2.2. Surface Hydrophobicity

Surface hydrophobicity reflects the distribution of hydrophobic amino acid residues on the protein’s surface, and characterizes the changes of tertiary structure of protein [30]. The surface hydrophobicity of oxidized WPI increased significantly (*p* < 0.05) with the AAPH concentration, the maximum achieved at 10 mmol/L AAPH, which is 84% greater than the control (Figure 1b), indicating that the peptide chains were moderately unfolded and rearranged [16], and the surface hydrophobic groups were exposed to the polar environment. This result corresponded to those of endogenous fluorescence; meanwhile, oxidized myofibrillar proteins from *Culter alburnus* also showed similar trends [18].

#### 3.2.3. Particle Size Distributions

**Figure 2:**
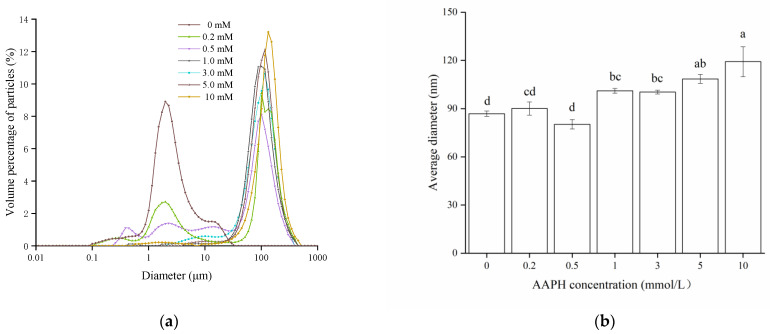
Particle size distributions (**a**) and average diameter (**b**) of WPI oxidized with different concentrations of AAPH. WPI = walnut protein isolates, AAPH = 2, 2′-azobis (2-amidinopropane) dihydrochloride. Different letters indicate significant differences between columns (*p* < 0.05).

Particle size is the common indicator to evaluate protein aggregation [31], and can be monitored by dynamic light scattering. Particle size distributions and average particle size of WPI oxidized with different concentrations of AAPH are shown in Figure 2. The particle size of control WPI was mainly distributed between 1–10 μm, while a large peak at 100 μm was observed after oxidation, illustrating formation of large aggregates through the interaction of protein peptides by oxidation [32]. Moreover, there is a rising trend on average particle size with higher AAPH concentration, with maximum values (119.2 µm) at 10 mmol/L AAPH, which is similar to the result of Fu et al. [16]. Overall, the above results suggested that the hydrophobic groups exposure (Figure 1b), as well as the disulfide and secondary bonds cross-linking (Table 1) might be responsible for the formation of larger protein aggregates.

#### 3.2.4. SDS-PAGE

**Figure 3:**
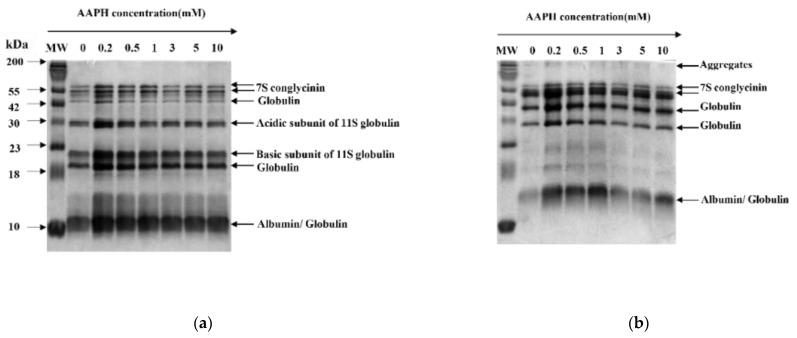
Sodium dodecyl sulphate–polyacrylamide gel electrophoresis (SDS-PAGE) patterns of WPI oxidized with different AAPH concentration (**a**) Reducing (**b**) Non-reducing. WPI = walnut protein isolates, AAPH = 2, 2′-azobis (2-amidinopropane) dihydrochloride; MW= molecular weight.

SDS-PAGE has the advantages of being fast, of high resolution and high sensitivity, and is especially suitable for the analysis and identification of oligomers and their subunits, as well as molecular weight determination of protein. As shown in the non-reducing SDS-PAGE pattern (Figure 3b), the intensity of 7 S conglycinin bands decreased with increasing AAPH concentration; meanwhile, new large molecule weight bands were observed at the top of the gel, indicating that large polymers may be formed by stronger oxidation [19]. Moreover, most of the degraded bands caused by oxidation were recovered in the reducing SDS-PAGE pattern (Figure 3a), and thus we reasoned that disulfide bond polymerization is the predominant manner for aggregates formation, which is similar to the study of Fu et al. [16].

#### 3.2.5. Fourier Transform Infrared (FT-IR) Spectroscopy

**Figure 4:**
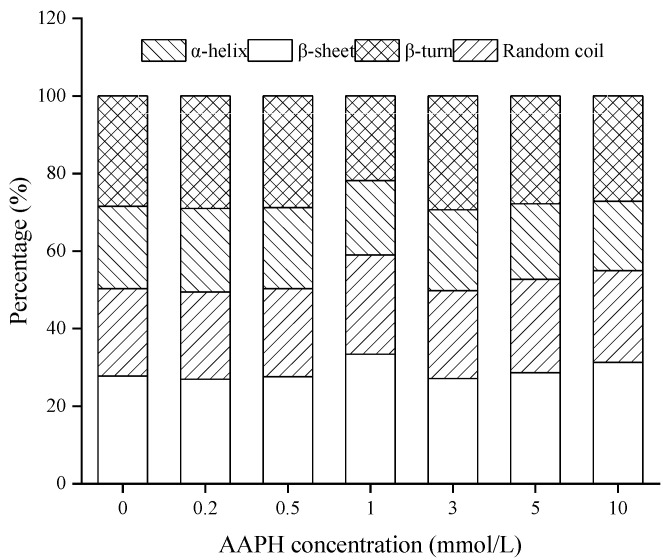
Secondary structure of WPI oxidized with different concentrations of AAPH. WPI = walnut protein isolates, AAPH = 2, 2′-azobis (2-amidinopropane) dihydrochloride.

The secondary structure of WPI could be reflected by the FT-IR spectrum, specifically, the amide 1 region was processed in de-convolution and second derivative, and the featured wavenumber of α-helix is 1648–1655 cm^−1^, β-sheet is 1610–1629 cm^−1^, β-turn is 1660–1681 cm^−1^, and random coil is 1638 cm^−1^ [24]. As shown in Figure 4, the percentage of α-helix decreased whereas that of β-sheet and random coil increased with the AAPH concentration. Studies have shown that α-helix is associated with the weak hydrogen-bond interaction between amino groups and amidogen, whereas β-sheet is related to the hydrogen-bond interaction between peptide chains [33]. Thus we deemed that the hydrogen-bond interaction between amino bond of WPI was broken by oxidation, and the unfolding peptide chains further re-associated to form a new construction (β-sheet and random coil), which in turn decreased the flexibility of the protein structure [28]. This finding is in accordance with the oxidized chickpea protein isolates reported by Zhu et al. [23].

### 3.3. Physicochemical Properties Analysis

#### 3.3.1. Solubility

Solubility is one of the critical characteristics of food proteins, and is often used to represent the degree of cross-linking and aggregation to a certain extent. As shown in Table 2, with the increasing AAPH concentration, the solubility of WPI shows a significant downward trend (*p* < 0.05), and the lowest solubility (20.44%) was observed at 10 mmol/L AAPH. This may be due to the unfolding and destruction of WPI conformation by peroxy radical oxidation, then further polymerization occurs to form macromolecule insoluble aggregates by covalent or non-covalent interactions [34]. The result of solubility is similar to that of myofibrillar protein from *Culter alburnus* oxidized by hydroxyl radical [18], and also echoes the result of particle size distribution in this study.

#### 3.3.2. Foaming Ability (FA) and Foaming Stability (FS)

Foaming property is an important interfacial property for protein processing, and is impacted by solubility, hydrophobicity, and flexibility of peptide chain. As indicated in Table 2, with the increase of AAPH concentration, the FA of WPI increased first and then decreased, and reached the maximum (36.67%) when the concentration of AAPH was 1 mmol/L. This is possibly because appropriate oxidation makes non-covalent bonds of WPI unfold, thereby the flexibility of the peptide chain increases. Consequently, more protein was adsorbed and formed a relatively stable gas–liquid interface. However, when WPI was over oxidized, the internal structure of the protein was largely unfolded and reconstructed, which led to the drop down of solubility markedly, and then a decline in FA. Meanwhile, FS also showed a similar trend by oxidation, and the maximum (83.33%) was identified at 1 mmol/L AAPH. The above research is similar to that on chickpea protein and rice bran globulin [23,24].

#### 3.3.3. Water Holding Capacity (WHC) and Oil Holding Capacity (OHC)

WHC and OHC are known to be linked to the water/oil binding ability of protein, and can significantly affect the texture of protein foods. As presented in Table 2, the WHC and OHC of oxidized WPI exhibited an initial increment followed by a decreasing tendency with AAPH concentration, and both the WHC (4.05 g/g) and OHC (5.84 g/g) reached maximum at 3 mmol/L AAPH. This is probably because, on the one hand, the hydrophobic groups of WPI were partly unfolded by oxidation, and some charged amino acid exposed to the surface, thus made the net charge of WPI increased [12]; and these mentioned changes of protein would be an advantage for WHC. On the other hand, macromolecular protein aggregation was formed by intermolecular disulfide bond crosslinking in oxidation [18], which would be a disadvantage for WHC. Therefore, the positive effects appear to be stronger than the negative effects in mild oxidation, and the water molecules entered the internal protein with non-covalent bonds, that made the WHC increase correspondingly. However, when the concentration was higher than 3 mmol/L AAPH, the negative effect was stronger than positive effects, thus decreasing the WHC dramatically. To the OHC, the oil adsorption capacity of WPI was enhanced with the hydrophobicity in certain concentrations of AAPH (≤3 mmol/L), and the OHC increased accordingly. Nevertheless, in strong oxidative conditions (>3 mmol/L), WPI was crosslinked and aggregated, which reduced the OHC consequently.

### 3.4. Proteomic Analysis of Oxidative Modified WPI

**Figure 5:**
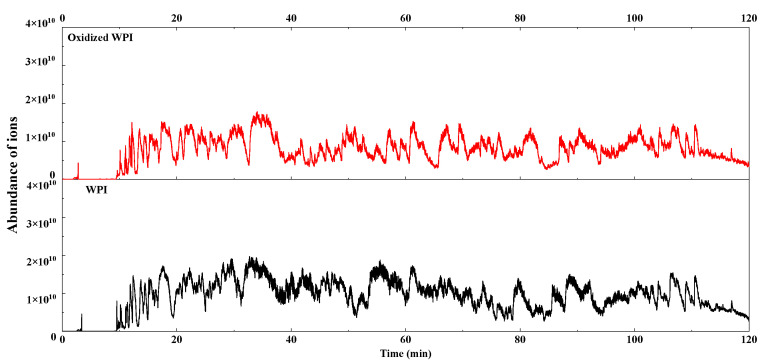
The total ion chromatogram (TIC) of WPI and oxidized WPI. WPI = walnut protein isolates.

In order to further investigate the impact of oxidation on the structure of WPI, a Nano LC-MS/MS peptide sequencing analysis was performed, and the total ion chromatograms (TIC) of unoxidized and oxidized protein were shown in Figure 5. Although the trend of the TIC plot of both samples seemed to be very similar, the differences in protein abundance still revealed changes.

By mass spectrometry analysis, a total of 3095 peptide spectra were detected, of which 646 protein groups were corresponding to 2396 unique peptide sequences (Supporting Information File S1, Table S1). According to their respective peptide sequences, the modifications in the functional group of the amino-acid side chains were analyzed and are listed in Table 3, including acetyl, carbamidomethyl, carboxyethylation, γ-glutamic semialdehydes (GGS), dehydrated, dehydroalanine, deoxidation, 4-hydroxy-2-nonenal (HNE), hydroxykynurenine, kynurenin, lactyllysine, malondialdehyde (MDA), oxidation, trioxidation, α-aminoadipic acid (AAA), and α-aminoadipic semialdehyde (AAS). For the different amino acids, cysteine, methionine, and tryptophan had the greatest number of side-chain modification types (5), followed by histidine, tyrosine and proline (4), and phenylalanine (3), whereas methionine and arginine created two types of modification. The above results are consistent with results previously reported on amino acids side-chains most prone to oxidative modification [35]. These modifications by oxidation may induce changes in the structure of the proteins, which in turn affected the function of proteins. For example, cysteine has unique reactivity which is associated with sulfhydryl reactive chemicals, hence, cysteine substitution has been a powerful tool to investigate the structure and function of proteins [36]. Methionine oxidation may influence the peptide hydrophobicity [37], where histidine residues in proteins are major targets for reaction with the lipid peroxidation product HNE [38], and so forth.

**Figure 6:**
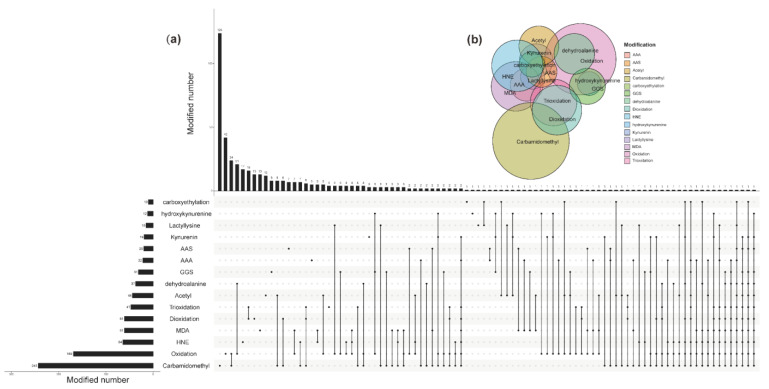
(**a**) UpSet plot of interactions between different modifications. (**b**) Venn diagram of interactions between different modifications. AAA = α-aminoadipic acid; AAS = α-aminoadipic semialdehyde; GGS = γ-glutamic semialdehydes; HNE = 4-hydroxy-2-nonenal; MDA = malondialdehyde.

The UpSet plot and Venn diagram were constructed to further visualize the numbers and intersections of the identified peptide from different modifications. From Figure 6, the top five modifications ranked by modified number were carbamidomethyl, oxidation, HNE, MDA, and deoxidation. They were 243, 169, 64, 61, and 61 times, respectively. Of them, 124 times occurred alone for carbamidomethyl, and 119 times were the combination of carbamidomethyl with other modifications. The Wayne diagram in the upper part of the figure also showed the same results. It was generally believed that proteins from an animal source were more susceptible to undergoing oxidative reactions than plant proteins, while GGS and AAS were the significant protein oxidative indicators in meat and dairy products [39]. The results of this study indicated that numerous modifications also occurred in oxidized WPI, whereas carbamidomethyl and oxidation was the most significant indicator.

**Figure 7:**
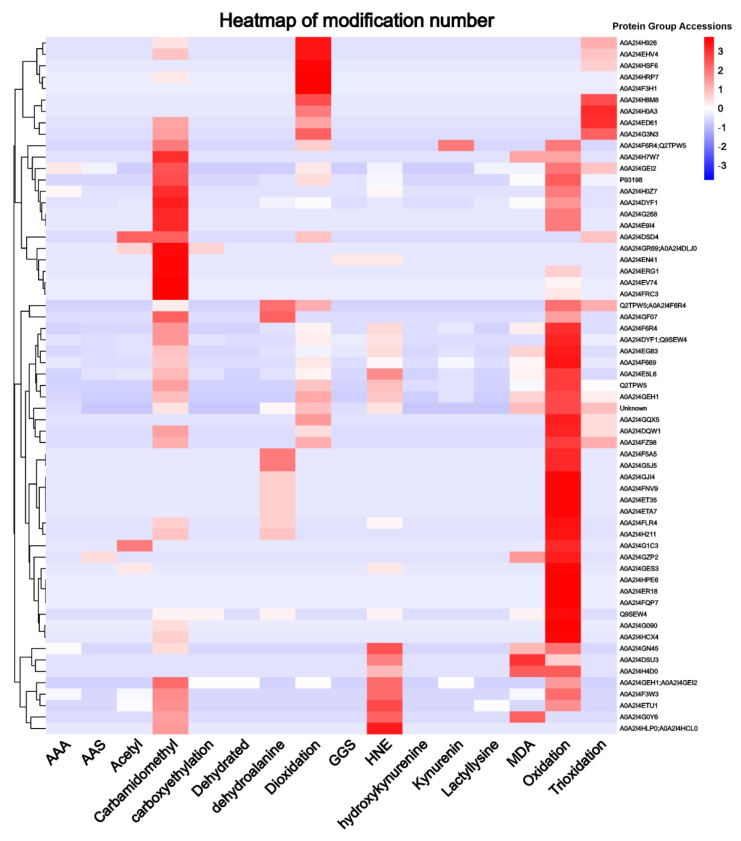
The heatmap of modification numbers. AAA = α-aminoadipic acid; AAS = α-aminoadipic semialdehyde; GGS = γ-glutamic semialdehydes; HNE = 4-hydroxy-2-nonenal; MDA = malondialdehyde.

To present the degree of modification on different protein groups more intuitively, a heatmap (Figure 7) was drawn in log10-transformed modification numbers (only total modification numbers equal to or greater than 5 are displayed). From Figure 7, oxidation, carbamidomethyl, deoxidation, HNE, and trioxidation were, in order, the most modified protein groups, and their modification numbers were greater than 5. This conclusion was basically consistent with the conclusion above. For different protein groups, the top 10 protein groups with the most modification numbers were P93 198 (190 times), A0A2I4F6R4 (127 times), A0A2I4DYF1 or Q9SEW4 (117 times), A0A2I4EG83 (108 times), Q2TPW5 (94 times), A0A2I4E5L6 (90 times), A0A2I4GEH1 (82 times), A0A2I4DYF1 (81 times), and Unknown (43 times). These proteins were mainly depicted as albumin seed storage protein, legumin-like protein, vicilin-like protein, and 11 S globulin seed storage protein. Interestingly, most of these proteins were found to be associated with allergies [40,41].

**Figure 8:**
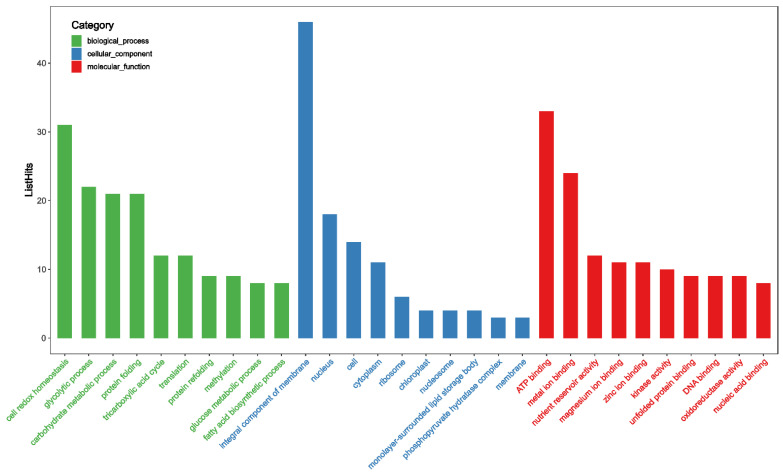
The top 10 functional enrichment analyses of gene ontology (GO).

Mass spectrometry of these peptides detected the presence of 519 proteins, and detailed information of these proteins is presented in Supporting Information File S2: Table S2. To further understand the functions of these proteins, gene ontology (GO) annotation was performed (Supporting Information File S3: Table S3). Through analysis, 378 GO terms were annotated, including 115 terms in biological process (BP), 204 in molecular function (MF), and 59 in cellular component (CC), and the top 10 enriched GO terms of different classifications are displayed in Figure 8. It can be seen clearly that “cell redox homeostasis” was the most enriched (number = 31) in BP, while the most enriched (number = 46) in CC was “integral component of membrane”. For MF, it was “ATP binding” with 33 enrichment numbers. Taking into consideration that protein modifications, especially post-translational modification, could regulate the structure and function of protein, including lifetime, assembly, localization, function, and degradation, it was therefore of great interest to further understand the main biological functions of these proteins [42].

### 3.5. Antigenicity of Jug r 1

**Figure 9:**
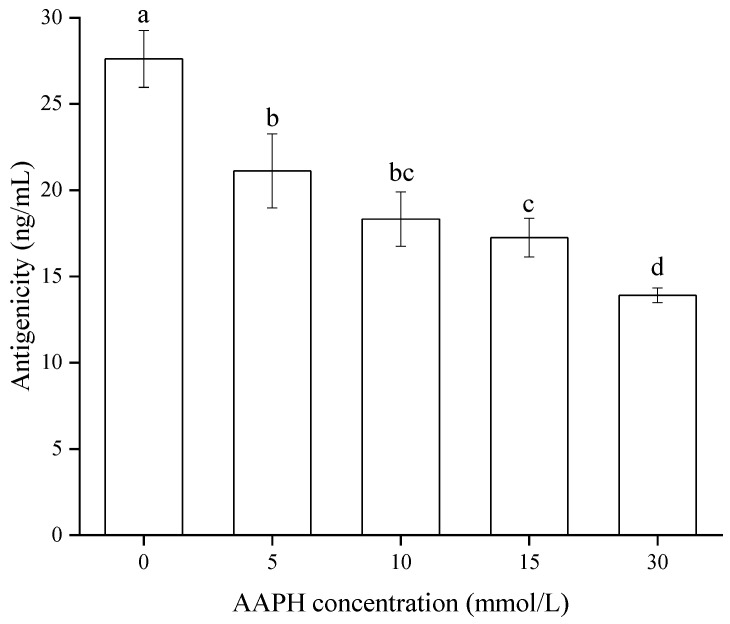
Effect of oxidation on antigenicity of Jug r 1. AAPH = 2, 2′-azobis (2-amidinopropane) dihydrochloride. Different letters within a column indicate significant differences (*p* < 0.05).

Jug r 1, which is 16.4 kD long and contains 139 amino acids, is the major allergen protein in walnuts, and could reflect the allergenic properties of WPI. As hinted at by Figure 9, the effect of oxidation on the antigenicity of Jug r 1 was assessed. It can be observed that there was a gradual decrease of antigenicity with the AAPH concentration, and the lowest value (13.91 ng/mL) was obtained at 30 mmol/L, indicating a significant effect (*p* < 0.05) on antigenicity by radical oxidation. Combined with the mass spectrometry results, changes in oxidative indicators and secondary structure (Table 1 and Figure 4), the oxidative modification of WPI might be covalent, and the changes of protein side chains led to the destruction of allergen epitopes, which consequently decreased the antigenicity of Jug r 1 [43]. In addition, the unfolding of WPI blocked the epitopes and could not be identified by an antibody. Similar results were exhibited in acrolein-oxidized shrimp tropomyosin [44] and hydroxyl radical-treated β-conglycinin [45], whereas the specific reason for the reduction of antigenicity should be further researched.

## 4. Conclusions

This paper explored the influence of peroxy radicals on structural and functional characters of WPI. The results showed that the carbonyl group of WPI was significantly increased with the concentration of AAPH, whereas the free amino and sulfhydryl groups decreased significantly (*p* < 0.05), which could be due to formation of disulfide bonds and irreversible sulfonic, sulfonic acid groups by radical oxidization. The reduction of fluorescence intensity and increasing of surface hydrophobicity illustrated the unfolding and rearranging of protein tertiary structure; meanwhile, FT-IR analysis also demonstrated alteration of the secondary structure. Besides, large aggregates were observed by particle size distributions and SDS-PAGE analysis, and thus unfolding and cross-linking of WPI were further confirmed. The aforementioned changes of conformation in turn affected the functional properties of WPI, and thus we saw a significant reduction in solubility, whereas different degrees of increase in foaming property and WHC/OHC were observed by mild oxidation. Nevertheless, all of the measured property indicators in this study were found to be significantly decreased after overoxidation. Mass spectrometry analysis revealed numerous modifications of the amino acid side chains, and these modifications are involved in many protein groups. Interestingly, these protein groups were found to be associated with allergies, which implied that oxidative modification could have an effect on the allergenic properties of WPI. The double antibody sandwich ELISA result indicated the gradual reduction of Jug r 1, the major allergen of WPI, and this further confirmed the mass spectrometry results. In conclusion, peroxy radical oxidation altered the conformation of WPI, which in turn caused modification in functional properties correspondingly, including allergenicity.

Proteins are one of the most important components of foods and are easily oxidized during their harvesting, storage, and processing. Although it is well known that protein structure strongly influences its functions, little is known about oxidative modifications and how it affects food protein structure and function. Generally, the preliminary properties, and secondary and tertiary structure of proteins can be investigated via multiple physical, chemical, and biological means, including a chromogenic assay, protein electrophoresis, spectroscopic studies, mass spectrometry, etc. However, they are often studied in isolation, and the results are incoherent. In this study, an integrated approach was used to produce results at multiple levels, seeking to provide a comprehensive understanding of the effect of oxidation induced by peroxy radical on the functional properties of WPI. Our results suggest that oxidation can lead to the modification of amino acids in proteins, subsequently altering their structure, which in turn changes their functional properties. Although the extent of protein modification is strongly increased with the increase of AAPH concentration, and it appears to perform adversely for the functional properties of the protein, appropriate oxidation is favorable for some properties, such as foaming property, WHC/OHC, and allergenicity. In conclusion, a potential methodology to limit oxidation, including antioxidants, should be taken into account. On the other hand, the above results also revealed the possibility of oxidation as a means to regulate protein functional properties in the future.

## Figures and Tables

**Table 1:** Carbonyl, free sulfhydryl, total sulfhydryl, and free amino groups of WPI oxidized with different concentration of AAPH.

AAPH (mmol/L)	Carbonyl (nmol/mg)	Free Sulfhydryl (nmol/mg)	Total Sulfhydryl (nmol/mg)	Free Amino (nmol/mg)
0.0	0.61 ± 0.08 c	6.66 ± 0.10 a	172.43 ± 16.17 ab	0.67 ± 0.07 a
0.2	0.85 ± 0.05 b	6.03 ± 0.21 b	173.09 ± 11.10 a	0.65 ± 0.05 ab
0.5	0.90 ± 0.10 b	5.11 ± 0.04 c	165.66 ± 4.59 ab	0.60 ± 0.03 b
1.0	0.93 ± 0.05 b	4.18 ± 0.13 d	155.70 ± 4.76 bc	0.54 ± 0.04 c
3.0	1.30 ± 0.15 a	3.16 ± 0.04 e	148.00 ± 3.69 cd	0.48 ± 0.03 d
5.0	1.42 ± 0.01 a	2.34 ± 0.04 f	135.64 ± 2.17 d	0.43 ± 0.02 e
10.0	1.48 ± 0.12 a	1.25 ± 0.09 g	114.26 ± 4.35 e	0.31 ± 0.04 f

WPI = walnut protein isolates, AAPH = 2, 2′-azobis (2-amidinopropane) dihydrochloride. Different letters within a column indicate significant differences (*p* < 0.05).

**Table 2:** Solubility, WHC, OHC, and foaming property of WPI oxidized with different concentration of AAPH.

AAPH (mmol/L)	Solubility (%)	WHC (g/g)	OHC (g/g)	FA (%)	FS (%)
0.0	80.64 ± 3.41 a	3.34 ± 0.05 b	4.55 ± 0.07 d	22.66 ± 3.65 e	72.22 ± 6.80 c
0.2	69.25 ± 3.72 b	2.61 ± 0.03 d	4.63 ± 0.08 d	27.78 ± 2.72 cde	74.58 ± 8.72 cb
0.5	59.65 ± 2.96 c	2.98 ± 0.15 c	4.87 ± 0.07 bc	28.89 ± 3.44 bcd	78.50 ± 5.48 abc
1.0	46.87 ± 2.41 d	3.31 ± 0.12 bc	5.00 ± 0.04 b	36.67 ± 3.65 a	83.33 ± 3.65 a
3.0	39.71 ± 3.07 e	4.05 ± 0.17 a	5.84 ± 0.19 a	33.33 ± 4.22 ab	81.39 ± 5.00 ab
5.0	31.48 ± 3.79 g	3.86 ± 0.15 a	4.74 ± 0.03 cd	31.11 ± 3.44 bc	78.33 ± 2.58 abc
10.0	20.44 ± 2.49 h	3.35 ± 0.29 b	4.64 ± 0.07 d	25.33 ± 2.98 de	72.36 ± 6.46 c

WPI = walnut protein isolates, AAPH = 2, 2′-azobis (2-amidinopropane) dihydrochloride; WHC = water holding capacity; OHC = oil holding capacity; FA = foaming ability; FS = foaming stability. Different letters within a column indicate significant differences (*p* < 0.05).

**Table 3:** The amino acid modified sites and types in the oxidation of WPI.

Amino Acid	Peptide Sequence	Modification Types												
		CAM	OX	DI	DHA	TRI	HNE	KYN	3-HK	GGS	CAE	ACE	AAS	AAA
Cysteine	**C**FDGSLFEY**C**AK	√												
Cysteine	GKLNFGRALE**C**FFLSSCSSPCFCMNSMESQDEFETR		√											
Cysteine	**C**FDGSLFEYCAK					√								
Cysteine	GLHGAAIPG**C**PETFQSESSSQFR			√										
Cysteine	M**CC**WNAPP**C**GFFYLNVDGAIFFYIHKAGVGAVVR						√							
Methionine	CQDE**M**R		√											
Methionine	QCCQQLSQ**M**DEQCQCEGLR			√										
Proline	CTNNAEKI**PP**GTVR			√										
Proline	CGDQVGC**P**CIGFDGLDVASECGPSCECGLECGNRSTQR		√											
Phenylalanine	**F**RPGTVALR		√											
Phenylalanine	NFYLAGNPDDE**F**RPQGQQEYEQHRR			√										
Phenylalanine	**F**MFWFACGVGGLEIICIFLVWCLLSR					√								
Histidine	**H**NLDTQTESDVFSR						√							
Histidine	ILRPVSVPG**H**FEAFHGSGGEDPESFYR			√										
Histidine	ILRPVSVPG**H**FEAFHGSGGEDPESFYR		√											
Histidine	QL**H**VILK					√								
Lysine	**K**EDIEMALTK						√							
Lysine	RVNALENVV**K**PR											√		
Lysine	**K**GSLGCIKYILK										√			
Lysine	VGESPSPATASS**K**PEQAR													√
Lysine	EAAYNLHLIY**K**												√	
Tyrosine	CDGCVRSIFPPF**Y**TCAQCGFFLHKSCVELSR		√											
Tyrosine	CLKLYCECFAAGI**Y**CVGSCACETCFNKPEYEDLVLDTR				√									
Tyrosine	ACGTCCARCDCVPPGTSGNYDACPC**Y**ANMTTHGGR			√										
Tyrosine	VVQCTEGERS**Y**HIFYQLCAGAPAALR					√								
Tryptophan	FVSVCYHNYEHVYCF**W**HRGMICVCCHT**W**PEILYYK		√											
Tryptophan	FLIGDDEHC**W**SENGVSNIEGGCYAK							√						
Tryptophan	FML**W**FACGVGGFEIICIFLVWCLLSR			√										
Tryptophan	ANRMG**W**FQLQR								√					
Tryptophan	GALYSDALYVPH**W**NLNAHSVVYALRGR					√								
Arginine	**R**ATGEGFEWVSFK									√				
Arginine	**R**QEEAEWEEEEAR						√							
Proline	ESFNLECGDVIRV**P**AGATVYVINQDSNERLEMVK			√										
Proline	ESFNLECGDVIRV**P**AGATVYVINQDSNERLEMVK	□	√	□	□	□	□	□	□	□	□	□	□	□

Note: Tick mark represents the corresponding modification was detected, the lower line and bold font represent the corresponding modification site. WPI = walnut protein isolates, CAM = carbamidomethyl; OX = oxidation; DI = dioxidation; TRI = trioxidation; DHA = dehydroalanine; HNE = 4-hydroxy-2-nonenal; KYN = kynurenin; 3-HK = 3-hydroxykynurenine; GGS = γ-glutamic semialdehydes; CAE = carboxyethylation; ACE = acetyl; AAS = α-aminoadipic semialdehyde; AAA = α-aminoadipic acid.

## Data Availability

The data presented in this study are available on request from the corresponding author.

## Supplementary Materials

The following supporting information can be downloaded at: https://www.mdpi.com/article/10.3390/foods11030385/s1, Table S1: peptide information; Table S2: protein information; Table S3: gene ontology (GO) annotation.
